# Scientific techniques in adolescent and young adult classic Hodgkin lymphoma

**DOI:** 10.1002/jha2.786

**Published:** 2023-09-29

**Authors:** Francesco Maura, Ragini M. Adams, Tomohiro Aoki

**Affiliations:** ^1^ Sylvester Comprehensive Cancer Center University of Miami Miami Florida USA; ^2^ Division of Pediatric Hematology, Oncology Stanford University School of Medicine Stanford California USA; ^3^ Princess Margaret Cancer Centre University Health Network Toronto Ontario Canada

## Abstract

Understanding the tumor microenvironment and genomic landscape is crucial for better prediction of treatment outcomes and developing novel therapies in Hodgkin lymphoma (HL). Recent advancements in genomics have enabled researchers to gain deeper insights into the genomic characteristics of HL at both single‐cell resolution and the whole genome level. The use of noninvasive methods such as liquid biopsies and formalin‐fixed paraffin‐embedded‐based imaging techniques has expanded the possibilities of applying cutting‐edge analyses to routine clinically available samples. Collaborative efforts between adult and pediatric group are imperative to translate novel findings into routine patient care.

## Graphical abstract

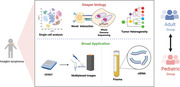

## 1 INTRODUCTION

Hodgkin Lymphoma (HL) represents the most common lymphoma subtype in people under the age of 30 [1]. Most patients affected by HL can be cured with modern treatment regimens including polychemotherapy and radiotherapy [2]. However, 25%–30% of patients will experience relapse or progression and eventually succumb to their disease [3]. A similar proportion might be over‐treated, leading to increased late toxicities and overall mortality in HL. Therefore, better prediction of treatment outcome has been a major research goal and novel therapeutic strategies are imperatively needed to improve survival.

HL is unique amongst virtually all cancers as the malignant Hodgkin and Reed Sternberg (HRS) cells are greatly outnumbered by reactive, non‐neoplastic cells in the tumor microenvironment (TME) [4]. HRS cells are typically found at low cellular frequency ranging from 0.1% to 10%. The HL TME mainly includes B and T cells, macrophages, eosinophils, and stromal cells. To date, the scarcity of the malignant HRS cells has hampered biological study in HL including genomic characterization. In this review, as part of review series associated with lymphoma research foundation (LRF) adolescent and young adult (AYA) Lymphoma consortium Workshop held in 2022, we comprehensively introduce cutting‐edge technologies and methodology, which help to overcome these limitations and facilitate recent biology studies in lymphoma.

## 2 GENOMICS OF REED STERNBERG TUMOR CELLS

Over the last 15 years, the development of next generation sequencing approaches has significantly expanded our understanding of cancer biology, identifying several new drivers and therapeutic targets [5, 6]. The application of these approaches to decipher the pathogenesis and genomic heterogeneity of HL have been historically challenging by the limited amount of Hodgkin and HRS tumor cells. Using optimized fluorescence‐activated cell sorting (FACS) or microdissection techniques, different groups were able to perform whole exome sequencing (WES) revealing mutations in critical driver genes involved in NF‐κB and JAK/STAT signaling pathways as well as genes involved in immune escape [7, 8, 9, 10]. However, WES is known to have a limited resolution on important genomic features such as structural variants, copy number changes and mutational signatures. To capture these key events and to comprehensively characterize the genomic landscape of HL, we interrogated whole genome sequencing (WGS) of FACS isolated HRS cells from 25 patients with HL including tumors from pediatric and adult patients [11]. Strikingly, despite the limitation in coverage (median ∼27X) and stringent criteria applied to correct for multiple rounds of DNA amplification, we observed a high mutational burden per sample (median 5279 SNV per WGS), particularly elevated among pediatric and adolescent and young adult (Ped/AYA). No association was found according to Ebstein‐Barr Virus (EBV) status, but the number of Ebstein‐Barr Virus (EBV)+ HL was limited. The high HL mutational burden was also suggested in whole exome studies, in particular among EBV‐ patients [10]. Interestingly, this increased mutational burden was driven mostly by aging single base substitution (SBS) signatures (SBS1 and SBS5) [12]. Despite the mechanisms behind the acceleration of clock‐like mutation rate are still unknown, the high HL mutational burden might be a key factor in promoting response to immunotherapies as observed in distinct solid tumors (e.g., PD1L and PD1 inhibitors) [13]. Looking at other mutational processes involved in HL genomic landscape, we also observed high prevalence of cases with APOBEC mutational activity (SBS2 and SBS13; 64%), and presence of SBS25 in patients exposed to ABVD‐like treatment, indirectly demonstrating the previously hypothesized direct mutagenesis of procarbazine/dacarbazine [12].

**FIGURE 1:**
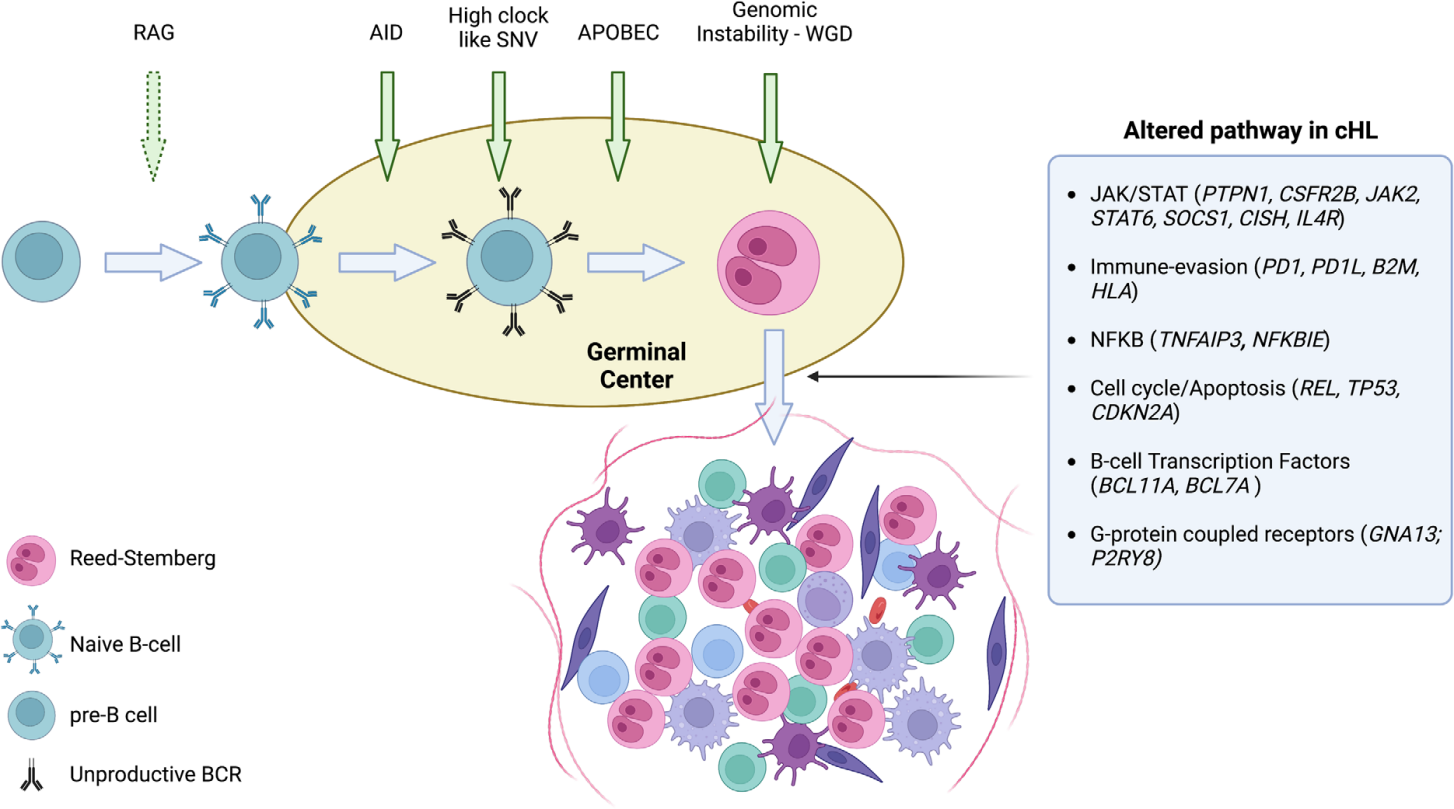
Cartoon summarizing the classic Hodgkin Lymphoma pathogenesis. The figure was generated using Biorender.

Combining this WGS series with 36 WES, we identified 26 genes significantly enriched for nonsynonymous single nucleotide variants (SNV) and indels and 19 recurrent copy number alterations (CNA) peaks (five gains and 14 losses) involving known and new HL genomic drivers. Importantly we confirmed HL high ploidy (median 2.95), often driven by whole genome doubling, observed in 59% of the patients. The integration of SNV/indels and recurrent CNA allowed the following observations: (1) we defined how distinct tumor suppressor genes are often involved by bi‐allelic inactivation; (2) distinct genes such as *SOCS1* have often more than one mutation per patients and this was driven by AID somatic hypermutation in the germinal center (GC); (3) looking at the relative timing of driver mutation acquisition with respect to chromosomal gains, we observed that SNV/indels in driver genes often proceed chromosomal gains and WGS in HL. The late acquisition of large chromosomal gains was also confirmed running molecular time analysis [14], which using the corrected ratio between duplicated and not duplicated mutations estimates the relative time in which large chromosomal gains are acquired in each patient life. Finally, WGS resolution further expanded HL genomic complexity reporting high prevalence of complex events such as chromothripsis (32%) and breakage‐fusion‐bridge (28%), often acquired before large chromosomal gains [15, 16, 17]. Despite the samples size, both single and complex SV events often involved important HL oncodrivers such as *PTPN1* (24%), *REL* (20%), *HLA‐B* (20%), *POU5F1* (20%). In addition to this finding, we also observed a significant enrichment of Recombination activating gene (RAG) motif in SV breakpoints [18], suggesting that some driver events can be acquired before the first GC encounter. Overall these data confirmed and expand the catalogue of genomic drivers involving the *JAK*/*STAT*, *NFKB*, and the interaction with the surrounding immune environment (Figure 1). Importantly, leveraging on the integration of Structural variants (SV), SNV, and Copy number variation (CNV) some of these drivers emerged as more prevalent than previous reported [8, 10, 19].

In conclusion, the identification of key genomic drivers through complex approaches such as FACS‐based HSR WGS emerged as a powerful approach to identify key genomic drivers and reconstruct the tumor life‐history. Considering this and previous WES‐based findings, it will be important to use and expand invasive and noninvasive (i.e., liquid biopsy) approaches to investigate the impact of these genomic features on clinical outcomes.

## 3 CIRCULATING TUMOR DNA AND LIQUID BIOPSIES

Precision medicine approaches in HL have, to date, been elusive largely due to an incomplete understanding of the genomic landscape of the disease and a dearth of robust biomarkers to guide escalation and de‐escalation of therapy. To address these shortcomings, we have developed several ultrasensitive techniques to characterize genotypic heterogeneity using circulating tumor DNA (ctDNA) in diverse tumor types [20, 21, 22], as well as novel methods to dynamically monitor disease risk [23, 24, 25]. Using these unique tools in combination with “liquid biopsies,” we aim to yield an unbiased view of the pediatric and AYA HL genomic landscape and to facilitate early, noninvasive assessment of therapeutic efficacy.

Liquid biopsies have advantages over traditional biopsies in that obtaining peripheral blood is minimally invasive, reflective of contributions from multiple tumor deposits, and can easily be repeated over time, allowing a more comprehensive analysis of tumor heterogeneity [26]. Importantly, unlike archival tumor tissue, cell‐free DNA (cfDNA) and its tumor‐derived subset (ctDNA) have not undergone fixation, and therefore constitute higher quality DNA facilitating high‐fidelity genotyping at low allelic fractions (AFs). We have successfully applied Cancer Personalized Profiling by deep Sequencing (CAPP‐Seq), a well‐established capture‐based targeted sequencing method, to 274 pretreatment plasma samples from HL patients [20, 27]. In addition to genome‐wide assessment of somatic CNA, we specifically designed a targeted capture panel spanning 615 kb of the human genome, targeting the full coding regions of 151 genes known to be recurrently mutated in B‐cell lymphomas including HL, as well as regions to detect structural variants and regions affected by Phased Variants. Interestingly, when comparing >300 HL blood and tumor tissue specimens by CAPP‐Seq, including matched tumor/plasma pairs (*n* = 17), we found a significantly higher malignant burden in the blood plasma than in tissue in most patients, allowing the noninvasive recovery of mutations. Surprisingly, despite the relative paucity of malignant cells in HL tumors, we found comparable levels of ctDNA per unit of PET metabolic tumor volume (MTV) in HL as observed in DLBCL, suggesting that liquid biopsy may potentially be as useful in HL as it is in DLBCL. Therefore, ctDNA genotyping of HL is likely to overcome limitations of tumor biopsy specimens, and to simultaneously capture the body tumor burden at baseline, during therapy, and after therapy.

In our discovery cohort of 44 pediatric and AYA patients, we detected at least 1 single nucleotide variant in 38 samples (86%). This is an indication that our methods are sensitive enough to reliably identify tumor‐related mutations in the vast majority of patients from a serum sample alone. When we looked at tumor genome equivalents per mL of plasma, we saw that baseline levels of ctDNA correlated with risk group, speaking to the potential utility of incorporating this measure into a baseline risk assessment. Further delineation of which threshholds are associated with increased risk is a focus of our ongoing work. Additionally, we interrogated our database of adult and pediatric patients with HL and found that variant allele frequencies are higher in younger patients, giving some insight into the biological differences between pediatric/AYA HL patients as compared to adult HL patients. A detailed understanding of how this disease is biologically different in older and younger patients is critical for appropriately tailoring therapy. The most commonly mutated gene in our cohort was TNFAIP3, which was detected in 45% of cases. Beta‐2 microglobulin and SOCS1 mutations were found in 42% of cases. While these are known genetic alterations in adult patients with HL, the recurrent mutations and their frequencies have never been comprehensively described in an exclusively pediatric and AYA population. Additionally, we have seen some early signals—delayed molecular response and rising ctDNA levels after therapy—which may be predictive of poor outcome in these patients, as we have shown in other cohorts. These early signals are an indication that ctDNA has the ability to differentiate out certain higher risk patients based on their detectable altered ctDNA kinetics.

Data from ctDNA can also be used in combination with imaging data to form a radiogenomic framework for predicting treatment failure in pediatric and AYA HL through an integrative strategy to combine tumor genotypes, ctDNA dynamics, and quantitative functional imaging responses. We recently described the Continuous Individualized Risk Index (CIRI) as a new statistical framework to dynamically determine outcome probabilities for individual patients integrating risk predictors acquired over time [24]. We will relate distinct genomic/molecular features and molecular subtypes, measures of tumor burden as well as dynamic response markers (ctDNA and Positron emission tomography (PET) 2) to key clinical outcomes, including early response (PET2 and ctDNA), event‐free (EFS), and overall survival (OS). We will then build a CIRI model specifically for young HL patients. This method dynamically determines outcome probabilities for individual patients by integrating risk predictors acquired over time as they become available [24].

In summary, the advantages of liquid biopsies in combination with the precision of our molecular techniques have the potential to address the critical unmet need to improve outcomes for patients with difficult‐to‐treat pediatric and AYA HL while minimizing toxicity following conventional chemotherapy and radiotherapy.

## 4 TUMOR‐MICROENVIRONMENT CHARACTERIZATION AT SINGLE CELL RESOLUTION

The emergence of novel drugs in the immunotherapy field including checkpoint inhibitors and chimeric antigen receptor (CAR) therapy in lymphoid cancers highlighted the importance to understand fundamental biological concepts of tumor‐microenvironment ecosystem in lymphoma. In particular, considering the highest response rate of the PD‐1 checkpoint inhibitors [28, 29, 30, 31, 32] in patient with HL among any cancer type [33], and their unique TME composition, HL could be the most exciting application fields of novel technology in the context of TME study. Historically, past studies have demonstrated biomarker potential of TME in HL [34, 35] using immunohistochemistry (IHC) by evaluating protein expression on either the tumor cells and/or the immune cells in the TME. More recently, the utility of bulk transcriptome sequencing data has been demonstrated to establish a gene expression profiling‐based prognostic model assay [36, 37, 38]. Especially, the Nanostring‐based gene expression profiling assay on the RNA extracted from formalin‐fixed paraffin‐ embedded tissue (FFPET) has shown reproducibility and biological validity. Indeed, the assay have contributed development of prognostic model in adult and pediatric HL [36, 37, 38]. However, these studies do not incorporate important information like comprehensive co‐expression pattern, cellular composition information and cell to cell spatial interactions to fully describe complex architecture of the TME.

Fortunately, recent technical advances have allowed for more comprehensive characterization of TME biology. For instance, single cell RNA‐sequencing (scRNA‐seq) analyses have contributed to facilitate deep understanding of biology of lymphoma by characterizing transcriptome state of TME at single cell level resolution [34, 39–49]. As a result, several research successfully revealed previously unknown diversity and rare subtypes in both cancer cells and immune cells in the TME. Multiplexed phenotyping approaches like mass spectrometry‐based detection by laser abrasion (imaging mass cytometry, IMC) and multiplexed ion beams imaging [50] also added new dimension for TME studies. Moreover, a combination of single cell RNA sequencing (scRNA‐seq) and multiplexed imaging technique allows for detailed functional and spatial characteristics of immune cells in the tumor microenvironment at a single‐cell resolution [34, 51]. As a first single cell sequencing study in HL, Aoki et al. applied droplet‐based single cell RNA‐sequencing of 22 HL and 5 reactive lymph node (RLN) samples. By applying phonograph algorithm, 22 distinct clusters were identified and enrichment of the regulatory T‐cell (Treg) population in HL was observed when compared with RLN. Further deep scRNA‐seq analyses revealed unique co‐expression patterns in cells of Treg cluster in the samples of HL, which includes strong expression of LAG3 along with other Treg markers such as GITR, CTLA4, and CD25. However, surprisingly, FOXP3, another canonical Treg marker, is not co‐expressed on these cells in HL. This phenotype is consistent with type 1 regulatory (Tr1) cell phenotype, which has immunosuppressive function through production of cytokines such as IL10 and TGFb. This is one of the prime examples that how single cell technology could help us to identify previously unrecognized population. Previous studies often utilize one representative Treg marker, FOXP3, to define Treg subset where Tr1 cells do not express.

Examination of spatial arrangement with IMC and subsequent functional analyses identified unique localization of LAG3+ Tr‐1 cells surrounding HRS cells and their negative interaction with major histocompatibility complex (MHC) class II on HRS cells. IMC showed that MHC class II negative HL cases have numerous LAG3+CD4+ T cells, with rare FOXP3+CD4+ T cells while MHC class II positive HL cases showed abundance of FOXP3+CD4+ T cells. Based on these findings, HL microenvironment composition can be classified into two or more distinct type according to MHC class II status on HRS cells [51], one of which is characterized by immune‐suppressive TME mainly induced and organized by LAG3 high Tr‐1 cells [51]. Considering the fact MHC class II positivity cells is associated with response of PD‐1 blockade in HL [52], LAG3 targeting strategy might be reasonable approach, especially for MHC class II negative HL. Of note, a recent paper in melanoma showed additive efficacy of dual checkpoint inhibition (LAG3 and PD‐1) in comparison to monotherapy (relatimab + nivolumab vs. nivolumab alone) [53], indicating the promising potential of dual targeting strategy.

Recent another scRNA‐seq study also identified unique CXCL13+ CXCR5 negative helper T cell subset in the TME of rare lymphocyte rich HL subset. These studies confirmed the robust power of single‐cell transcriptome profiling to identify putative cell‐cell interactions in lymphoma. From technology perspective, as a next step, single cell multiomic studies including single cell RNA and ATAC sequencing or single cell DNA and RNA sequencing will provide an integrated multi‐layered approach to understand lymphoma biology, including in special populations like AYAs lymphoma, where there can be limited access to tissue samples. Future work aimed at continuing to decipher the crosstalk between malignant and non‐malignant cells holds the promise to accelerate rapid development of new biomarkers and therapeutic strategies.

## 5 CONCLUSION

Recent technological advancement provides us new opportunities to study biology of lymphoma at unprecedented resolution. As a next step, integration of these technologies and/or multi‐omics analysis approach would further facilitate our deep understanding of biology of HL. Moving forward, while exciting new technologies will boost comprehensive biology study, we need to establish the strategy to translate these novel findings into routine patient care to improve patient outcome. Further collaborative efforts are imperative to facilitate biomarker translation, in particular, rare pediatric and young adult lymphoma study. In that context, the effort of organizing conferences and workshops focusing on specific fields would play important role to facilitate collaborative opportunity towards these goals. The application of FFPET and plasma samples, which are easily available samples in daily clinical practice, would aid to expand collaborative genomic and TME study in both real worlds setting and in clinical trial setting. Particularly, the biomarker value of newly identified immune cell populations and ctDNA will need to be confirmed in the context of prospective trials. In that way, we could further boost biology‐driven risk adopted targeted treatment to improve treatment outcome while minimizing toxicity.

## AUTHOR CONTRIBUTIONS

F.M., R.A., and T.A. reviewed the literature and wrote the paper.

## CONFLICT OF INTEREST STATEMENT

The authors declare no conflicts of interest.

## PATIENT CONSENT STATEMENT

The authors have confirmed patient consent statement is not needed for this submission.

## CLINICAL TRIAL REGISTRATION

The authors have confirmed clinical trial registration is not needed for this submission.
